# Exploring the relationship between resilience, sense of coherence, and social support in a sample of nurses during the spread of COVID-19: a mediation analysis study

**DOI:** 10.3389/fpubh.2024.1451236

**Published:** 2025-01-17

**Authors:** Camelia Rohani, Mehrnaz Ahmadi, Maryam Seyedtabib, Ladan Mehdipoorkorani

**Affiliations:** ^1^Department of Health Care Sciences, Palliative Care Research Center, Marie Cederschiöld University, Stockholm, Sweden; ^2^Department of Community Health Nursing, School of Nursing & Midwifery, Shahid Beheshti University of Medical Sciences, Tehran, Iran; ^3^Nursing Care Research Center in Chronic Diseases, Medical and Surgical Nursing Department, School of Nursing and Midwifery, Ahvaz Jundishapur University of Medical Sciences, Ahvaz, Iran; ^4^Department of Biostatistics and Epidemiology, School of Public Health, Ahvaz Jundishapur University of Medical Sciences, Ahvaz, Iran; ^5^Medical and Surgical Nursing Department, Taleghani Hospital, Ahvaz Jundishapur University of Medical Sciences, Ahvaz, Iran

**Keywords:** resilience, sense of coherence, social support, nurse, COVID-19, Iran

## Abstract

**Background:**

During the spread of COVID-19, nurses on the front line of fighting the disease experienced severe psychological pressures. The ability to adapt to difficult situations is an essential requirement for maintaining individuals’ endurance during a crisis. This study was designed to explore the relationship between three outcome variables of resilience, sense of coherence (SOC), and perceived social support in a sample of Iranian nurses during the spread of the COVID-19 pandemic.

**Methods:**

A cross-sectional study with a correlational design was conducted on 360 registered nurses from six university hospitals during the seventh wave of COVID-19 in our society. Data collection was conducted by the demographic information questionnaire, the Connor–Davidson Resilience Scale (CD-RISC), the Sense of Coherence Scale (SOC-13), and the Multidimensional Scale of Perceived Social Support (MSPSS).

**Results:**

The results of the structural equation modeling (SEM) showed that social support had a direct positive effect on the SOC (*β* = 0.498, *p* < 0.001), and the SOC had a direct positive effect on the resilience (*β* = 0.688, *p* < 0.001). Furthermore, we found an indirect effect of social support on resilience through the complete mediating role of the SOC.

**Conclusion:**

The full mediating role of SOC in the relationship between social support and resilience showed that nurses with a higher level of SOC had a better understanding of social support, and this can influence their resilience in the COVID-19 crisis. This is theoretical support for the application of the salutogenic approach to health intervention plans to promote a healthy orientation in nurses toward mobilizing resources.

## Introduction

Now more than 4 years have passed since the beginning of the COVID-19 pandemic worldwide, but its harmful psychological effects on the general health of individuals have remained (1). Nurses, as the first group on the front line of facing the COVID-19 pandemic, experienced different severe psychological pressures (2). Continuous stress experienced by nurses—stemming from high workloads, the fear of transmitting the coronavirus to family and friends, feelings of loneliness, low perceived social support, and decreased life expectancy—has resulted in numerous physical, mental, social, and spiritual challenges for them (1–3). The results of a study conducted to examine nurses’ perceptions and experiences during the COVID-19 outbreak in Iran revealed that Iranian nurses experienced high levels of psychological distress. This distress was attributed to several factors, including the unexpected nature of the situation, insufficient knowledge and skills to manage it, and concerns about the health of their families, as well as social stigma (4). Furthermore, they brought consequences such as weakness in decision-making and prioritization of care and ultimately a decrease in the quality of patient care (5).

Evidence indicates that the ability to adapt to challenging situations, known as resilience, has been a crucial factor in maintaining individuals’ endurance during crises such as COVID-19 (6, 7). Resilience is regarded as a personality trait that enhances one’s capacity to explain and predict issues and increase the capacity to cope with stressful events (8). Individuals with high resilience possess the ability to strategize, make sound decisions, and try to solve problems by operational and communication skills. They have a positive attitude toward themselves and their activities (9). The results of earlier studies indicate that higher resilience among nurses during the COVID-19 pandemic was strongly associated with increased job satisfaction, improved quality of patient care, reduced burnout, and lower levels of psychological distress (6, 10–12). Various factors influence the resilience of nurses, among them sense of coherence (SOC) as an inner strength can be a useful factor in dealing with unavoidable stressors. Previous literature shows that higher SOC was related to higher resilience (13). In other words, SOC appeared to be beneficial for mental health and mental well-being strengthening the individual’s resilience (14, 15).

SOC is an individual’s overall orientation to his life, and it guides him to find and use appropriate resources to maintain his health and manage his stress (16). It has been raised in Antonovsky’s Salutogenic model. Antonovsky defined the SOC as a stable and dynamic sense in humans that makes life events comprehensible, manageable, and meaningful (17). SOC is an inner strength that moderates the effects of stress on health (16). Nurses who have high levels of SOC are more task-oriented and are more capable of dealing with tensions in their work environments (18). Studies conducted during the COVID-19 pandemic have indicated that nurses confronted many challenges, and the SOC is indicated to be a strong predictor of psychological components and mental health (19). Thus, nurses with a low SOC experienced more psychological distress (2).

In addition to the SOC, another factor that plays a predictor of mental health and leads to the improvement of the level of resilience in individuals is social support (3, 20). Perceived social support (PSS) includes love, empathy, care, self-esteem, attention, and receiving help from others and refers to people’s cognitive evaluation of relationships and support systems (20). It is one of the components that plays a significant role in nurses’ performance in critical situations and plays a significant role in overcoming stress and tensions in the nursing work environment (12, 21). Low levels of social support in nurses have been associated with job burnout, depression, stress, anxiety, and reduced performance in the care of their patients (3, 22). The results of studies during the COVID-19 pandemic have also indicated the positive effects of social support on reducing psychological pressures caused by the pandemic (3).

Considering the psychological needs and problems of nurses during the COVID-19 pandemic and the importance of paying attention to this category of issues, healthcare sector employees, especially nurses, need to be equipped with the necessary psychological skills (23). For this purpose, the correct understanding of the psychological needs and problems of individuals is very important. It is also necessary to identify strategies that can protect the mental health of nurses after the COVID-19 pandemic and/or other crises. These strategies enable them to have an appropriate response to crises in the future (24). It is important to know that the COVID-19 pandemic is not over yet and it will not be the last pandemic in our lives. Thus, it is always important to deal with crises and be aware of practical solutions.

Although there are various studies in the field of resilience and its relationship with the variables of social support and SOC, and also some studies have been published recently that have investigated these three variables simultaneously (25, 26), the aims of these studies are different from ours, and the effect of these three variables on each other has not been investigated. We tried to explore the relationship between resilience, SOC, and social support in a sample of Iranian nurses during the spread of the COVID-19 pandemic. By identifying the direct, indirect, and general effects of these variables on each other, the mechanisms of how the variables affect each other would be clarified (27).

## Methods

### Design

This is a cross-sectional study with a correlational design which was conducted from the first of September 2021 to the end of January 2022, simultaneously with the seventh wave of COVID-19 in our society (28).

### Hypotheses

To better understand the relationships between resilience, SOC, and PSS, we propose the following hypotheses:

1. The level of PSS has a direct positive effect on the level of resilience.
2. The level of PSS has a direct positive effect on the level of SOC.
3. The level of SOC has a direct positive effect on the level of resilience.
4. The level of PSS has an indirect effect on the level of resilience through the level of SOC (the mediating role of the SOC).

### Sampling and data collection

Registered nurses were selected by quota sampling from six major university hospitals in Ahvaz, the capital of Khuzestan province, Southwestern Iran. Nurses who worked at least 6 months in COVID-19 time at university hospitals with a minimum of 1 year of nursing experience were employed to participate in the study. According to the nurses’ population at university hospitals (*n* = 1900), 320 nurses were enrolled in the study. Calculation of the sample size was conducted by the Cochrane formula (95% confidence interval and 80% power of the study). We accessed the list of nurses’ names through the nursing departments of the university hospitals and selected eligible nurses. One and a half times the calculated quota in each university hospital, an online invitation letter to participate in the study together with an online informed consent form and a link to the website to access the questionnaires was sent to eligible nurses through social networks and/or SMS. They were asked to answer the questionnaires for four working days. In this way, 540 registered nurses in six university hospitals got these invitations, but only 366 registered nurses answered positively to participate in the study (67.8%).

### Measurements

Data were collected through a demographic information questionnaire and a Persian version of the three self-reported scales.

#### Demographic information questionnaire

The questionnaire included variables such as age, sex, education level, work experience, and history of caring for COVID-19 patients.

#### Connor–Davidson resilience scale

The resilience of nurses was measured using the CD-RISC. The CD-RISC consists of 25 items and 5 subscales: personal competence, high standards and tenacity (8 items), trust in one’s instincts, tolerance of negative affect and strengthening effects of stress (7 items), positive acceptance of change and secure relationships (5 items), self-control (3 items), and spiritual influences (2 items). The responses are rated on a 5-point Likert scale ranging from completely incorrect (score: 0) to completely correct (score: 4). The values on the scale range from 0 to 100, with higher values, indicating greater resilience (29). A Persian version of the CD-RISC has been validated in Iran (30). The reliability of the scale was calculated at 0.83 using Cronbach’s alpha coefficient in the present study.

#### Sense of coherence scale

Nurses’ SOC was measured by the SOC scale with 13 items and three subscales of comprehensibility (5 items), manageability (4 items), and meaningfulness (4 items). The scale has a semantic differential format with two anchoring responses (1 to 7). The scoring range is 13–91; the higher the score, the stronger the SOC (17). This scale has been previously validated in the Iranian population (31). In the current study, the reliability of the scale was calculated at 0.74 using Cronbach’s alpha coefficient.

#### Multidimensional scale of perceived social support

The MSPSS includes 12 items with three subscales: family (4 items), friends (4 items), and significant others (4 items) (32). The participants’ responses were rated on a 7-point Likert scale ranging from very strongly disagree to very strongly agree (1 to 7). The total score of the scale varies from 12 to 84, with a higher score, indicating better PSS. The scale has been validated in Iran (33). In the current study, the reliability of the scale was calculated at 0.96 using Cronbach’s alpha coefficient.

### Statistical analysis

**Figure 1:**
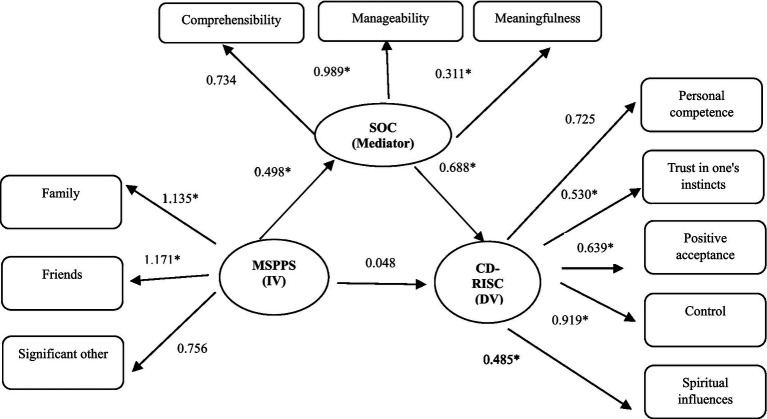
Structural equation modeling with dependent (DV) and independent (IV) variables. The numbers are standardized regression weights (*β*); SOC, sense of coherence; CD-RISC, Connor–Davidson resilience scale; MSPSS, multidimensional scale of perceived social support; *p < 0.001.

Data analysis was performed by SPSS version 20 (IBM Corporation, Armonk, NY, United States). All variables met the normality assumptions by P–P plots. An independent *t*-test was used to compare the mean score of resilience, the SOC, and the PSS in terms of dichotomous demographic variables. At first, relationships between three outcome variables (resilience, SOC, and PSS) were evaluated in a correlation matrix using Pearson’s correlation coefficients. Structural equation modeling (SEM) analysis was conducted by AMOS version 20.0 (34). All independent variables were included in the model, regardless of their significance, to understand the role of all variables. The effects of independent variables are adjusted in the model (coefficients in Figure 1). No paths have been excluded from the model. In the SEM analysis, maximum likelihood estimation (MLE) was applied as the most commonly used estimation method (35). Several specific indices and cutoff points were selected for SEM analysis. The indices were chi-square to the degrees of freedom ratio (criteria: ratio < 6), goodness-of-fit index (GFI) (criteria: ≥ 0.90), normed fit index (NFI) (criteria: ≥ 0.90), comparative fit index (CFI) (criteria: ≥ 0.90), Tucker–Lewis index (TLI) (criteria: ≥ 0.90), and incremental fit index (IFI) (criteria: ≥ 0.90), root mean square error of approximation (RMSEA) (criteria: less than 0.05 are good, between 0.05 and 0.08 are acceptable) (36). As a rule of thumb (37), the degree of SOC is considered a total mediator if the correlation between the independent (PSS) and dependent (resilience) variables would be non-significant in the SEM analysis, while their relationship was significant earlier in a two-by-two matrix.

## Results

### Descriptive results

The mean age of registered nurses was 33.6 ± 6.03 years old. Most of the nurses were females (74.1%) and 63.5% of them had a history of caring for COVID-19 patients in ICUs (30.5%) or general wards of university hospitals (33%). The history of caring for COVID-19 patients was significant in registered nurses based on the mean scores of three outcome variables. Nurses who had experience caring for COVID-19 patients had significantly higher resilience (*p* = 0.001), SOC (*p* < 0.001), and PSS (*p* = 0.001). The rest of the demographic characteristics of registered nurses is presented in Table 1 based on the mean levels of resilience, SOC, and PSS.

**Table 1:** Demographic characteristics of registered nurses based on the three outcome variables; the CD-RISC, the MSPSS, and the SOC (*n* = 366).

Variables	Number (%)	Total CD-RISC score (M ± SD)	Total MSPSS score (M ± SD)	Total SOC score (M ± SD)
Age (year)				
23–33	170 (46.4)	49.32 ± 13.58	52.07 ± 18.26	54.91 ± 10.24
≤34	196 (53.6)	52.08 ± 13.41	57.59 ± 17.41	56.97 ± 9.5
***p*-value**		0.05	0.003	0.04
Sex				
Female	274 (74.1)	51.21 ± 13.28	55.71 ± 17.21	56.67 ± 9.59
Male	92 (24.9)	49.58 ± 14.27	52.98 ± 19.93	54.05 ± 10.55
***p*-value**		0.32	0.24	0.03
Education level				
BScN	296 (80.9)	50.71 ± 13.57	54.69 ± 17.99	55.91 ± 10.01
MSN	70 (19.1)	51.15 ± 13.49	56.44 ± 17.82	56.42 ± 9.43
***p*-value**		0.80	0.46	0.69
History of caring for COVID-19 patients				
No	131(35.8)	47.70 ± 13.07	50.87 ± 17.42	53.19 ± 10.14
Yes	235 (64.2)	52.52 ± 13.51	57.34 ± 17.86	57.59 ± 9.41
***p*-value**		0.001	0.001	<0.001
Nursing experience (year)				
≤10	249 (68)	49.94 ± 13.41	53.37 ± 18.28	55.57 ± 9.92
≥11	117 (32)	52.63 ± 13.68	58.56 ± 16.76	56.94 ± 9.81
***p*-value**		0.07	0.01	0.21
Duration of working in the COVID-19 department (month)				
<12	182 (77.4)	52.57 ± 13.33	57.38 ± 18	57.89 ± 9.2
≥12	53 (22.6)	52.37 ± 14.26	57.22 ± 17.5	56.54 ± 10.13
***p*-value**		0.92	0.95	0.36
Employment status				
Permanent	217 (59.3)	52.42 ± 13.59	56.77 ± 17.15	56.87 ± 9.53
Temporary	149 (40.7)	49.68 ± 13.42	53.83 ± 18.42	55.42 ± 10.11
***p*-value**		0.05	0.12	0.16
Type of hospital				
General	184 (49.7)	50.87 ± 13.55	55.14 ± 17.98	56.02 ± 9.88
Specialized	182 (49.2)	50.73 ± 13.56	54.92 ± 17.96	56.01 ± 9.93
***p*-value**		0.91	0.90	0.99

BScN, bachelor of science in nursing; MSN, master of science in nursing; CD-RISC, Connor–Davidson resilience scale; MSPSS, multidimensional scale of perceived social support; SOC, sense of coherence.

Nurses’ mean scores of resilience with five subscales, the PSS with three subscales, and the SOC scale with three subscales are presented in Table 2. The mean scores of three outcome variables of resilience, PSS, and SOC were reported as 50.80 ± 13.54 (range: 0–100), 55.03 ± 17.95 (range: 12–84), and 56.01 ± 9.89 (range: 13–91), respectively (Table 2).

**Table 2:** Descriptive data for outcome variables, including the CD-RISC, the MSPSS, and the SOC in nurses (*n* = 366).

Outcome variables	Mean ± SD	Scale range
**Total CD-RISC**	50.80 ± 13.54	0–100
Personal competence	15.04 ± 5.67	0–32
Trust in one’s instincts, tolerance of negative affect	15.67 ± 3.47	0–28
Positive acceptance of change	10.37 ± 3.10	0–20
Control	5.90 ± 3.49	0–12
Spiritual influences	3.80 ± 1.91	0–8
**Total MSPSS**	55.03 ± 17.95	12–84
Family	19.01 ± 5.04	4–28
Friends	18.04 ± 6.68	4–28
Significant other	17.96 ± 7.16	4–28
**Total SOC**	56.01 ± 9.89	13–91
Comprehensibility	20.18 ± 5.98	4–28
Manageability	17.72 ± 3.41	4–28
Meaningfulness	18.11 ± 2.74	5–35

CD-RISC, Connor–Davidson Resilience Scale; MSPSS, Multidimensional Scale of Perceived Social Support; SOC, sense of coherence.

### Results of the two-by-two relationships between variables

Analyses started with the calculation of Pearson correlation coefficients between SOC, PSS, and resilience scale scores in a correlation matrix (Table 3). The results showed that there is a significant positive correlation between the PSS and the resilience (*r* = 0.51, *p* < 0.001) as well as between the SOC and the resilience (*r* = 0.61, *p* < 0.001). Furthermore, a significant positive correlation was found between the SOC and the PSS (*r* = 0.51, *p* < 0.001).

**Table 3:** Correlation matrix between variables of the CD-RISC, the MSPSS, and the SOC in a sample of Iranian nurses (*n* = 366).

Variables	SOC	Comprehen	Manage	Meaning	CD-RISC	PC	TIOI	PAO Ch	Control	SI	MSPSS	Family	Friends	Significant other
**SOC**	1	0.91^**^	0.86^**^	0.52^**^	0.61^**^	0.48^**^	0.38^**^	0.49^**^	0.55^**^	0.38^**^	0.51^**^	0.61^**^	0.49^**^	0.38^**^
Comprehen	0.91^**^	1	0.72^**^	0.23^**^	0.52^**^	0.41^**^	0.33^**^	0.42^**^	0.46^**^	0.29^**^	0.40^**^	0.47^**^	0.39^**^	0.30^**^
Manage	0.86^**^	0.72^**^	1	0.30^**^	0.63^**^	0.49^**^	0.38^**^	0.52^**^	0.60^**^	0.38^**^	0.49^**^	0.64^**^	0.48^**^	0.33^**^
Meaning	0.52^**^	0.23^**^	0.30^**^	1	0.29^**^	0.24^**^	0.18^**^	0.18^**^	0.23^**^	0.28^**^	0.35^**^	0.37^**^	0.31^**^	0.31^**^
**CD-RISC**	0.61^**^	0.52^**^	0.63^**^	0.29^**^	1	0.87^**^	0.76^**^	0.65^**^	0.84^**^	0.49^**^	0.51^**^	0.61^**^	0.47^**^	0.42^**^
PC	0.48^**^	0.41^**^	0.49^**^	0.24^**^	0.87^**^	1	0.47^**^	0.39^**^	0.68^**^	0.45^**^	0.47^**^	0.51^**^	0.45^**^	0.41^**^
TIOI	0.38^**^	0.33^**^	0.38^**^	0.18^**^	0.76^**^	0.47^**^	1	0.41^**^	0.71^**^	0.17^**^	0.36^**^	0.40^**^	0.33^**^	0.31^**^
PAO Ch	0.49^**^	0.42^**^	0.52^**^	0.18^**^	0.65^**^	0.39^**^	0.41^**^	1	0.39^**^	0.32^**^	0.27^**^	0.46^**^	0.18^**^	0.18^**^
Control	0.55^**^	0.46^**^	0.60^**^	0.23^**^	0.84^**^	0.68^**^	0.71^**^	0.39^**^	1	0.18^**^	0.48^**^	0.54^**^	0.45^**^	0.40^**^
SI	0.38^**^	0.29^**^	0.38^**^	0.28^**^	0.49^**^	0.45^**^	0.17^**^	0.32^**^	0.18^**^	1	0.26^**^	0.32^**^	0.27^**^	0.17^**^
**MSPSS**	0.51^**^	0.40^**^	0.49^**^	0.35^**^	0.51^**^	0.47^**^	0.36^**^	0.27^**^	0.48^**^	0.26^**^	1	0.90^**^	0.96^**^	0.96^**^
Family	0.61^**^	0.47^**^	0.64^**^	0.37^**^	0.61^**^	0.51^**^	0.40^**^	0.46^**^	0.54^**^	0.32^**^	0.90^**^	1	0.80^**^	0.81^**^
Friends	0.49^**^	0.39^**^	0.48^**^	0.31^**^	0.47^**^	0.45^**^	0.33^**^	0.18^**^	0.45^**^	0.27^**^	0.96^**^	0.80^**^	1	0.91^**^
Significant other	0.38^**^	0.30^**^	0.33^**^	0.31^**^	0.42^**^	0.41^**^	0.31^**^	0.18^**^	0.40^**^	0.17^**^	0.96^**^	0.81^**^	0.91^**^	1

** Correlation is significant at the 0.01 level (two-tailed).

Comprehen, Comprehensibility; Manage, manageability; Meaning, meaningfulness; CD-RISC, Connor–Davidson Resilience Scale; MSPSS, Multidimensional Scale of Perceived Social Support; SOC, sense of coherence; PC, personal competence; TIOI, trust in one’s instincts; PAO Ch, positive acceptance of change; SI, spiritual influences.

### Results of the role of the variables in the relationships

According to the results of the correlation matrix, the mediating role of the SOC was tested on the relationship between variables of PSS (independent variable) and resilience (dependent variable) by SEM analysis. After controlling for demographic variables of age (continuous variable), sex (dichotomous variable), education level (two categories), years of nursing experience (continuous variable), duration of working in the COVID-19 department (continuous variable), employment status (two categories), history of caring for COVID-19 patients (dichotomous variable), and type of university hospital (two categories), the level of SOC showed a fully mediating effect in the relationships between the PSS and the resilience. For clarity, independent variables have been omitted from Figure 1.

The fit indices of the model were not ideal before the modifications; however, after adjusting the relationships between covariances, the indices in the final modified model showed significant improvement (Table 4).

**Table 4:** Goodness of fit indices in hypothetical and modified models by SEM analyses in a sample of Iranian nurses (*n* = 366).

Model fit indices	X^2^, *p*-value	𝐱^2^ /df	GFI	NFI	IFI	TLI	CFI	RMSEA
Hypothetical model	696.995, *p* < 0.001	6.168	0.848	0.838	0.860	0.785	0.858	0.119
Modified model	351.309, *p* < 0.001	3.346	0.910	0.918	0.941	0.903	0.940	0.080

GFI, Goodness-of-fit index; NFI, normed fit index; IFI, incremental fit index; TLI, Tucker–Lewis index; CFI, comparative fit index; RMSE, root mean square error of approximation.

Therefore, in response to the hypotheses of the study, the first hypothesis was rejected (the direct positive effect of the PSS on resilience), but the rest of them were accepted (the direct positive effect of the PSS on the SOC and the direct positive effect of the SOC on the resilience). Moreover, the indirect effect of PSS on resilience through the level of SOC was confirmed.

The paths and standardized regression coefficients (the standardized regression weights represent the amount of change in the dependent variable that is attributable to a single standard deviation unit’s worth of change in the predictor variable) and the mediating effect of the SOC have been shown in the modified model in Figure 1. As shown, the PSS has a significant direct effect on the SOC (*β* = 0.498, *p* < 0.001), and the SOC has a significant direct effect on the resilience (*β* = 0.688, *p* < 0.001), but the direct effect between the PSS and the resilience (*r* = 0.51, *p* = 0.01 in Table 3) disappeared through the mediating role of the SOC (*β* = 0.048, *p* = 0.055) (Figure 1).

## Discussion

The present study is designed to test the relationships between three outcome variables of resilience, SOC, and PSS in a sample of registered nurses at university hospitals during the seventh wave of COVID-19 in Iran. Our results showed that the hypothetical model of the study had a good fit after adjustment and that all the hypotheses of this model were accepted except the first hypothesis (the level of PSS has a direct positive effect on the level of resilience). Thus, the complete mediating role of SOC in the relationship between PSS and resilience was confirmed in this model.

Previous studies showed that there is a bidirectional relationship between PSS and resilience. PSS refers to people’s subjective assessment of supportive relationships and behaviors (20) and is known as one of the most important external factors affecting people’s capacity to overcome stressful situations and their resilience level in difficult situations (38, 39). On the other hand, the level of resilience is also a person’s confidence in their coping abilities, self-esteem, and personal characteristics, which increases the reception of PSS from others (40). Because resilience emphasizes the use of coping strategies in difficult situations (8), it leads individuals to receive more PSS. In fact, due to their positive thinking, high sense of empathy, and cooperative problem-solving in unfavorable and tense situations, people who have high resilience will rush to help family members, friends, and others and enjoy a wide circle of support and social relations (3). Furthermore, studies have shown that nurses who received support from friends, family members, relatives, managers, and colleagues during the critical situation of COVID-19 reported higher resilience (41, 42).

According to our results, it can be argued that there was not a simple relationship between PSS and resilience among Iranian nurses during the conditions of the COVID-19 crisis. This relationship was influenced by an important salutogenic factor, i.e., nurses’ level of SOC. It seems that nurses’ salutogenic view of life in the form of the SOC promoted their healthy attitudes toward the use of available resources such as social support, and it can influence their resilience in their lives, probably caring for COVID-19 and non-COVID-19 patients and managing their stressors during the COVID-19 crisis. Focusing on the role and power of the nurses’ SOC on the relationship between their PSS and resilience is one of the great lessons learned during the COVID-19 epidemic. Planning for salutogenic interventions based on the level of SOC as an internal resource and its development can assist the nurses in overcoming daily stressors during the crisis. Although Antonovsky believed that SOC is developed until the age of 30 and then remains stable and only decreases slightly during crises (16), later studies showed that an individual’s SOC can be improved by health interventions (43). People probably develop and enhance their use of SOC strategies as they accumulate experiences in managing life over time and in different circumstances (44). Antonovsky (17), in his model, explained that the level of SOC is a salutogenic factor that creates positive health. Furthermore, the level of SOC is dependent on two types of resistance resources: generalized (GRRs) and specific (SRR). GRRs have a “wide-ranging” benefit to stress management, but SRRs have a “situation-specific” benefit on special occasions of stress management (16). PSS is a GRR that can assist in developing a stronger SOC (45). Interestingly, evidence shows that PSS, in comparison to the received social support, is linked to health outcomes (46). Thus, individuals’ perception of available social support can change their perspective on their lives and even lead them to have a higher SOC, more resilience, and health. In the impact of social support in stressful situations, two models were reported in the literature: “buffering social support” and “main social support.” In the first model, the people who perceive more social support experience less stress in stressful situations compared to those who have less PSS. In the second model, there is a perception of social support even without stressful conditions (47). It is a complicated process; however, it is more likely nurses experienced the buffering effect of social support during the COVID-19 pandemic in the present study. Findings from a qualitative study conducted during the COVID-19 pandemic to determine nurses’ perceptions of social support indicated that nurses engaged with the crisis to assess their social support requirements and the feasibility of meeting those needs. They concentrated on the support that was accessible or believed to be accessible while setting aside certain needs that could not realistically be met at that time. Support from colleagues and the organization played a critical role in this process (48).

In any case, the effect of PSS on resilience is activated in the presence of SOC. In other words, the way nurses look at their life events is important; how much they understand them is meaningful, manageable, and comprehensible, influencing the relationship between their perception of social support and resilience. In a review of the literature, we could not find the mediating effect of SOC in the relationships between variables of social support and resilience. However, the results of a similar study showed that SOC and social support were simultaneously predictors of resilience in nurses during the COVID-19 era, although SOC was introduced as the strongest predictor of resilience (25). Furthermore, previous studies showed that the level of SOC was shown to have a mediating effect on relationships between stress management and individuals’ perception of health and well-being in different samples (47, 49).

Jiang and Luo (49) studied the mediating role of SOC in the relationship between PSS and social anxiety and found that in addition to having a direct effect on reducing students’ social anxiety, the perception of PSS exerted an indirect effect through the mediation of SOC. This relationship shows that by strengthening SOC in people, it is possible to help resolve existing tensions and create greater effects on improving people’s mental health and resilience. SOC has three characteristics, namely perceptibility, controllability, and meaningfulness, which means that people with higher SOC perceive life experiences as more perceivable, controllable, and meaningful (17). The high level of these components in SOC reflects the fact that people have a higher ability to mobilize resources and facilities in critical situations and can use useful resources to reduce pressure in external environments to maintain their internal stability in social interactions. This leads to optimism and adaptability in behavioral domains and can cause more PSS from people around the individual. Therefore, individuals with higher SOC also receive higher perceived PSS (49).

PSS, SOC, and resilience are individuals’ ways of handling stressors and maintaining health and well-being (2, 21, 50). From the complex relationships between them, it can be concluded that the application of a salutogenic approach to health-targeted intervention (e.g., training programs or workshops) plans may promote a healthy orientation in nurses toward mobilizing resources and experiencing more social support to manage daily stressors and benefit more resilience during the COVID-19 pandemic.

### Strengths and limitations of the study

One of the strengths of the study is the investigation of relationships among three variables; such as resilience, SOC, and social support together. However, these relationships were evaluated in this study by a cross-sectional design. Therefore, for a deeper understanding of the causal relationships between these variables, it is suggested to evaluate them in longitudinal studies. In addition, the use of validated scales and the diversity of the sample from multiple hospitals can be as other strengths of this study.

## Conclusion

The relationships between the three outcome variables of resilience, SOC, and PSS in a sample of registered nurses during the seventh wave of COVID-19 in Iran showed that the level of SOC completely mediates the relationship between PSS and resilience. In other words, nurses with higher levels of SOC experienced better perceptions of social support, which in turn helped promote nurses’ resilience during the COVID-19 pandemic. Our results provide theoretical support to reveal the mechanism between these three variables during the COVID-19 crisis. It can serve as a hypothesis for future research to design studies based on salutogenic interventions to promote the level of SOC as well as PSS and implement longitudinal studies to show broader dimensions of the complex mechanism between these variables.

## Data Availability

The raw data supporting the conclusions of this article will be made available by the authors, without undue reservation.
